# Results of the use of human platelet-rich fibrin membrane applied on colorrhaphy in nourished and malnourished rats

**DOI:** 10.1590/acb397824

**Published:** 2024-11-08

**Authors:** Fábio Henrique de Oliveira, Lucas Silva de Oliveira, Luciana Xavier Pereira, Vinicius Silva Belo, Fernanda Elias Ferreira Rabelo, Guilherme Santos Couto, Antonio Lacerda-Filho

**Affiliations:** 1 Universidade Federal de São João del-Rei – São João del-Rei (MG) – Brazil.; 2 Centro Federal de Educação Tecnológica de Minas Gerais – Divinópolis (MG) – Brazil.; 3 Universidade Federal de Minas Gerais – Belo Horizonte (MG) – Brazil.

**Keywords:** Platelet-Rich Fibrin, Wound Healing, Colon, Sutures

## Abstract

**Purpose:**

To evaluate the effects of platelet-rich fibrin (PRF) on the healing of intestinal sutures in rats.

**Methods:**

Forty rats were distributed into four groups. Two groups were treated with a standard diet and considered nourished (I and II). Two other groups were treated with cornmeal and considered malnourished (III and IV). All animals underwent cecotomy and cecorrhaphy. Groups II and IV had sutures overlapped with human PRF membrane. The following parameters were evaluated: animal weight, death, rupture site, rupture pressure, collagen, and reticulin dosage in the suture line.

**Results:**

The use of PRF did not influence deaths, rupture pressure or rupture location. For malnourished animals, a significant difference was observed in relation to the rupture site, corresponding to the suture line (*p* = 0.038) and reticulin dosage (*p* = 0.040), when PRF was used. There was no difference in relation to burst pressures.

**Conclusions:**

The use of PRF did not influence intestinal healing in nourished rats. In the group of malnourished animals, its use favored healing.

## Introduction

The most feared complication in intestinal surgery is anastomotic dehiscence, which can occur in 2 to 15% of operated patients and is closely related to failure in the healing process. This complication has a high morbidity and mortality rate, interferes with surgical results, increases hospital costs, and in oncological cases worsens the prognosis. Several factors can influence the healing of the colonic anastomosis, such as colon preparation, surgical technique, nutritional status, tension in the suture line, use of manual or mechanical suture, infection, and use of pharmacological agents^1^^–^ 3.

Although there is insufficient evidence that nutritional supplementation helps wound healing, adequate nutrition is imperative for preventing infection, which has deleterious effects on healing^4^. In some situations, malnourished individuals may require intestinal procedures, and new strategies have been developed with the aim of improve the healing process.

One of the most promising are tissue substrates, which corroborate a favorable outcome, reestablishing the integrity of the structures, perhaps shortening the scar maturation time^5^. Platelet-rich fibrin (PRF) is a derivative of platelet-rich plasma, in which autologous platelets and leukocytes are present in a complex fibrin matrix that can be used to accelerate the healing of soft and hard tissue injuries. PRF is a concentrate of fibrin, which is an essential element for healing. The greater availability of fibrin increases the amount of collagen, a substrate that in greater quantities in malnourished animals can improve healing.

PRF was described in France by Dohan et al.^6^, and it was a substance approved by the Food and Drug Administration, of the United States of America. Despite the many studies published on its applicability in dentistry and other areas, there are few studies on the use of PRF in intestinal sutures, just as there are few studies that have evaluated the use of PRF in nourished and malnourished animal models^7^^–^ 10.

Therefore, this study aimed to evaluate the effects of the PRF membrane on colorraphy in nourished and malnourished rats, by evaluating the location and rupture pressure and measuring collagen and reticulin in the intestinal suture line.

## Methods

### Experimental design

Forty male Wistar rats (*Rattus norvegicus*) were used and allocated into two groups with 20 animals each, according to nutritional status:

- Group A: nourished animals;
- Group B: malnourished animals.

The animals were received for the experiment at 21 days old and already weaned. Water was offered without restrictions. Group A (nourished) was fed on demand with standard rodent food with a crude protein concentration of 22%. Group B (malnourished) was fed exclusively with cornmeal with a protein composition of 3.7%.

**Figure 1:**
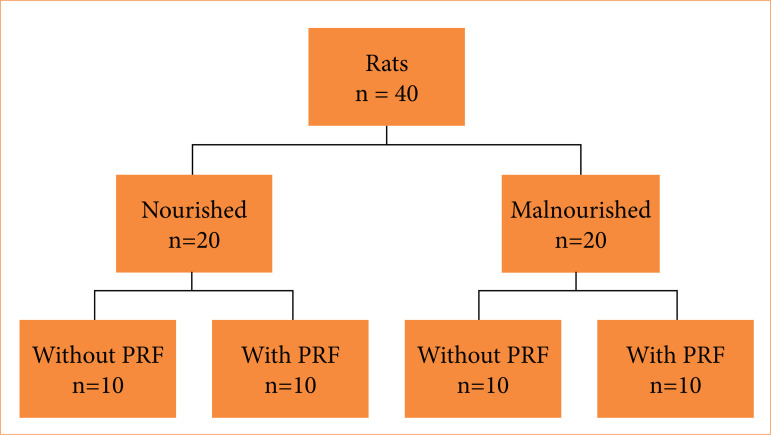
Allocation of animals into groups and subgroups according to the treatment performed.

The two groups were randomly divided into two other subgroups, which differed by the use of PRF, thus determining:

- Group I: nourished rats submitted only to intestinal suture (n = 10);
- Group II: nourished rats submitted to suture with PRF positioning (n = 10);
- Group III: malnourished rats submitted to suture only (n = 10);
- Group IV: malnourished rats submitted to suture with PRF positioning (n = 10) (Fig. 1).

PFR: platelet-rich fibrin. Source: Elaborated by the authors.

### Anesthetic and surgical procedure

The animals were anesthetized with xylazine hydrochloride at the dose of 5 mg/kg body weight intramuscularly and ketamine hydrochloride at the dose of 25 mg/kg body weight also intramuscularly. During the transoperative period, additional doses of anesthetic were administered, as needed, when muscle contractures or painful stigmata occurred. A 2-cm incision was made on the anti-mesocolic border of the cecum wall. The colorraphy was performed with continuous suture, with 5.0 polypropylene thread, with a 1.3-cm cylindrical needle. In animals in groups II and IV, in addition to this procedure, a 2-cm PRF membrane was positioned covering the entire colonic suture line, configuring an artificial patch. It was fixed with a continuous suture using 5.0 polypropylene thread.

The synthesis of the abdominal wall, in all groups, was performed in two planes:

- One including the peritoneum, muscle and aponeurosis, with simple continuous suture;
- The subcutaneous tissue and skin, with continuous suture, using thread nylon 3.0 with 1.5-cm cutting needle.

### Platelet-rich fibrin production

The PRF was prepared by collecting 10-mL samples of human blood from five volunteer donors who had signed the informed consent form. The samples were obtained before the surgical procedures by puncturing the veins in the cubital fossa of the volunteer donor and placed in a glass centrifuge tube without anticoagulant. The tubes were immediately placed in the centrifuge and centrifuged at 1,920 rpm for 10 min. Blood collection and transfer to the centrifuge should be as quick as possible, because, without anticoagulant, blood samples begin to clot instantly. If the time is longer than a few minutes, the fibrin will polymerize diffusely, and failure will occur. If the technique is correct, the platelets should be massively trapped in the fibrin meshes.

Three centrifugation layers were obtained: a fibrin clot in the middle of the tube, immediately between the red blood cells at the bottom, and the acellular plasma at the top.

**Figure 2:**
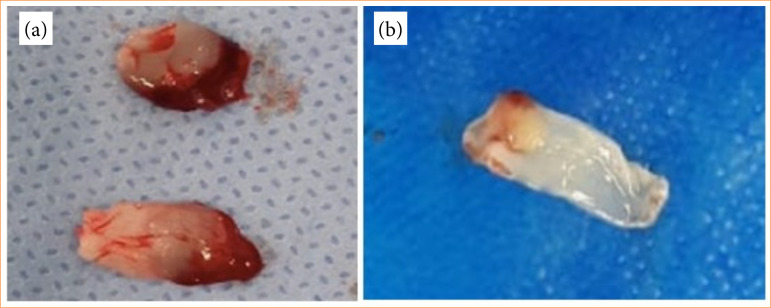
PRF manipulation. **(a)** Appearance of the platelet-rich fibrin before pressing; **(b)** Final appearance of the platelet-rich fibrin ready for use.

Source: Elaborated by the authors.

The upper straw-colored layer was removed, and the middle fraction was collected. A fibrin membrane was then obtained by removing the serum and the clot. The polymerization of fibrin in PRF is very slow and natural during centrifugation. Therefore, a structurally flexible, elastic, and strong membrane is formed. This material was then separated and pressed against a smooth surface with holes to obtain only the functional portion of the membrane (Fig. 2).

### Euthanasia and resection of the right colon

The animals were reoperated on the seventh postoperative day, after anesthesia with a dose five times greater than in the initial surgery, with the aim of causing euthanasia. A median xiphopubic incision was made, which allowed complete visualization of the abdominal cavity. The presence of signs of peritonitis, abscess, anastomotic leak, or intestinal obstruction was investigated. The right colon was sectioned, as far as possible from the suture line.

Immediately before anesthetic induction, all animals had their weight measured, as well as immediately after euthanasia, on an electronic scale.

### Anastomotic burst pressure study

The anastomotic rupture pressure was measured using an electromechanical device. This device consists of a linear spindle-based movement structure that continuously moves the syringe plunger, ensuring constant torque and speed throughout the movement. The equipment has a dedicated differential pressure sensor and microcontroller system, enabling data acquisition and storage at a sampling rate of 0.1 second, with a maximum measurement error limited to a band of ± 2.5% at full scale. These characteristics ensure the sensor’s measurement (calibration) for operation at 0 to 85°C. The procedure to measure the rupture pressure consisted of the following steps: the syringe was loaded with water, and an 8 Fr Nelaton probe was attached to the surgical specimen evaluated. As the plunger advanced, the sample was filled with water until rupture was identified. Simultaneously, the pressure in the set (syringe and sample assembly) was constantly measured.

The surgical specimen obtained was cleaned with saline solution with minimal pressure. The stump was gently adapted to the metal end of the probe and ligated with catgut 2.0. The maximum pressure reached at the moment of rupture was recorded.

### Assessment of the rupture site

A video device with a slow-motion system was used to define the location of the rupture defining it as at the suture line or outside the suture line. Possible anastomotic leaks were also tested in the same way.

### Histopathological study

Animals from groups III and IV were included in the histopathological analysis. The same segment submitted to previous analyses was prepared and fixed in 10% formaldehyde. The specimens were sent to the laboratory for the preparation of paraffin blocks. Sections were made perpendicular to the suture area of the cecum and stained with hematoxylin and eosin.

The healing assessment was carried out using light microscopy, by an experienced pathologist who was unaware of which group of animals the material belongs. The following indicators were considered in each specimen:

- Greater area of collagen close to the suture line at the highest magnification (40X);
- Greater area of reticulin close to the suture line at the highest magnification (40X).

Gomery staining was used to evaluate collagen; no differentiation was made between types I and III. The surgical specimens were analyzed along the suture line, and the quantification of the largest area containing collagen in this region was measured.

For reticulin, a specific staining was used to highlight reticulin (HistoKit). It was decided to define the largest stained area per animal, both for the subgroup without PRF and for the one with PRF.

The slides prepared for collagen and reticulin analysis, when analyzed at the highest magnification, 40X, presented a more striking and strong coloration. The stained area was quantified.

### Ethical aspects

The experimental procedures were carried out in accordance with the guidelines of the Brazilian College of Animal Experimentation. The research protocol was approved by the Animal Use Ethics Committee of the Universidade Federal de São João del-Rei, under number 018/2018, and by the Ethics Committee of the Universidade Federal de São João del-Rei (4552997). It was registered in Brazil Platform of Research, under protocol CAAE 39125920.8.0000.5545.

### Statistical analysis

The results obtained were analyzed using Statistical Package for the Social Sciences 20.0 and Microsoft Excel software. The sample calculation was based on parameters estimated from a similar previous study, with virtual online calculator (https://pt.surveymonkey.com/mp/sample-size-calculator). Comparison between groups was performed using analysis of variance for the variables of anastomotic rupture pressure and rupture location. In the malnourished group, collagen and reticulin levels in the suture line were also analyzed. Fisher’s exact test was used to compare the weights of animals between the nourished and malnourished groups. The Mann-Whitney’s U test was used to evaluate suture rupture pressure, amount of collagen and reticulin. The paired samples Student’s t test was used to compare the weight and hydroxyproline concentration on the days of surgery and reoperation. The χ^2^ test was used to evaluate the rupture site. Statistically significant differences were considered when values were *p* < 0.05.

## Results

Among the 40 animals in the study, four died (two from group III and two from group IV). The events occurred during the preoperative feeding period, and the ages of the animals varied between 64 and 68 days old.

It was observed that in the group with no PRF membrane there was dehiscence in two animals, three ruptures outside the suture line and three in the suture line. In the group in which the PRF membrane was used, in addition to no dehiscence, all rupture sites occurred outside the suture line (*p* = 0.038) (Table 1).

**Table 1:** Malnourished animals and location of rupture.

Break	Without PRF			With PRF		*p*-value
	n	%		n	%	
Dehiscence	2	25.00		0	0	0.038
Outside the suture	3	37.50		8	100	
At the suture line	3	37.50		0	0	

*χ^2^ test significant at 5%; PFR: platelet-rich fibrin. Source: Elaborated by the authors.

A significant association was observed in all assessments. In the group of malnourished animals, most rupture events were outside the suture line (78.57%), inferring greater fragility of the colonic wall in animals with nutritional deficiencies.

When the nourished groups were compared with the malnourished group that used PRF coverage, in the first group, all ruptures were at the suture line. In the second group, the ruptures were outside the line (*p* = 0.01). When the nourished and malnourished groups that did not receive PRF coverage were compared, it was observed that for nourished animals all ruptures were in the suture line. In the malnourished group, half of ruptures occurred on suture line (*p* = 0.036) (Table 2).

**Table 2:** Location of rupture by nutritional status.

Break	Group					*p*-value
	Nourished			Malnourished		
	n	%		n	%	
Outside	0	0		11	78.57	< 0.001*
Line	20	100		3	21.43	
**Break**	**Without PRF**					***p*-value**
	**Nourished**			**Malnourished**		
	**n**	**%**		**n**	**%**	
Outside	0	0		8	100	< 0.001*
Line	10	100		0	0	
**Break**	**With PRF**					***p*-value**
	**Nourished**			**Malnourished**		
	**n**	**%**		**n**	**%**	
Outside	0	0		3	50	0.036*
Line	10	100		3	50	

* Fisher’s exact test significant at 5%;

PFR: platelet-rich fibrin. Source: Elaborated by the authors.

The distribution of ruptures by type of treatment and by subgroup was analyzed. When animals with and without PRF were evaluated, a statistically significant difference was observed only for the groups of malnourished animals, in which the group with PRF showed a higher proportion of ruptures outside the suture line (Table 3).

**Table 3:** Rupture location with and without PRF.

Break	Treatment					*p*-value
	Nourished			Malnourished		
	n	%		n	%	
Outside	8	44.4		3	18.8	0.154
Line	10	55.6		13	81.2	
**Break**	**Nourished**					***p*-value**
	**Nourished**			**Malnourished**		
	**n**	**%**		**n**	**%**	
Outside	0	0		0	0	-
Line	10	100		10	100	
**Break**	**Malnourished**					***p*-value**
	**Nourished**			**Malnourished**		
	**n**	**%**		**n**	**%**	
Outside	8	100		3	50	0,07
Line	0	0		3	50	

PFR: platelet-rich fibrin. Source: Elaborated by the authors.

The initial weight of the fed animals that did not receive PRF ranged from 373 to 440 g (average of 417.4 g), and the weight at the time of euthanasia was from 358 to 420 g (average of 399.5 g). The animals that received PRF varied in weight from 352 to 535 g (average of 475 g) preoperatively. At the end of the experiment, the oscillation was between 336 and 514 g (average of 450.8 g). It was observed that for both groups the pre-operative weight was greater than the weight after the procedure (*p* < 0.001) (Table 4).

**Table 4:** Weight values of fed animals.

Group	Average	SD	Min.	Q1	Median	Q3	Max.	*p*-value
**Without PRF**								
Pre-weight	417.4	24	373	396	426.5	439	440	< 0.001*
Post-weight	399.5	21.3	358	384	406.5	414	426	
**With PRF**								
Pre-weight	475	56.4	352	443	493	512	535	< 0.001*
Post-weight	450.8	59.1	336	398	476	494	514	

* *p* < 0.05;

PFR: platelet-rich fibrin; SD: standard deviation; Min.: minimum; Max.: maximum. Source: Elaborated by the authors.

The malnourished group that did not receive PRF showed weight gain with an initial median of 87 g and a final median of 123 g. The malnourished group that received the PRF membrane also showed an increase in the median with an initial value of 94.3 g and final 133 g (*p* = 0.413). On the other hand, the subgroup of fed animals showed weight loss when comparing the initial and final weights. The median of the group without PRF ranged between 426.5 and 406.5 g. In the group with PRF, the median varied between 493 and 476 g (*p* = 0.008). The other results can be seen in Table 5.

**Table 5:** Weight variation by group before and after procedure.

Group	Treatment	Weight (g)							*p*-value
		Initial				Finale			
		Average	SD	Median		Average	SD	Median	
Malnourished	Without PRF	88.8	10.5	87.0		121.8	16.6	123.0	0.002**
Malnourished	With PRF	94.3	9.6	92.0		132.9	14.5	133.0	0.002*
	p value	0.413				0.245			
Nourished	Without PRF	417.4	24.0	426.5		399.5	21.3	406.5	0.016**
Nourished	With PRF	475.8	56.4	493.0		450.8	59.1	476.0	0.008*
	p value	0.008*				0.043*			

* Mann-Whitney’s test significant at 5%;

** Wilcoxon test significant at 5%;

PFR: platelet-rich fibrin; SD: standard deviation. Source: Elaborated by the authors.

The rupture pressure of the colic sutures in the 20 fed animals varied between 128 and 289 mmHg, with an average of 190 mmHg. No statistically significant difference was observed between the groups without PRF and with PRF in relation to burst pressure (*p* = 0.248) (Table 6).

**Table 6:** Bursting pressure (mmHg) in the group of fed animals.

Pressure	Average	SD	Min.	Q1	Median	Q3	Max.	*p*-value
Without PRF	180.1	47.0	127.7	153.0	171.9	194.9	287.0	0.248
With PRF	201.3	52.3	127.6	173.8	197.1	233.9	289.3	
Overall	190.7	49.6	127.6	159.9	177.7	227.9	289.3	

PFR: platelet-rich fibrin; SD: standard deviation; Min.: minimum; Max.: maximum. Source: Elaborated by the authors.

**Figure 3:**
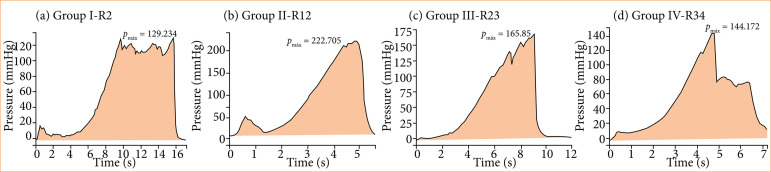
Graphic documentation.

Source: Elaborated by the authors.

Figures 3a and 3b present the curve that evaluates the burst pressure for the group of fed animals and show the exact moment and pressure of this event.

Figures 3c and 3d present the curve that evaluates the burst pressure for the group of malnourished animals and show the exact moment and pressure of this event.

The rupture pressure of the colic sutures in the 20 malnourished animals varied between 11.63 and 298.89 mmHg, with a mean of 157.5 mmHg, a median of 173.1 mmHg and a standard deviation of 112.1. In the subgroup of animals that received PRF, values ranged between 118.89 and 242.58 mmHg, with an average of 186.4 mmHg, median of 193.8 mmHg and standard deviation of 44.3 (Table 7).

**Table 7:** Bursting pressure in the group of malnourished animals.

Pressure	Average	SD	Min.	Q1	Median	Q3	Max.	*p*-value
Without PRF	157.5	112.1	11.6	46.5	173.1	255.2	299.0	0.867
With PRF	186.4	44.6	118.9	154.0	193.8	223.0	242.6	
Overall	171.0	85.8	11.6	118.9	193.8	242.6	299.0	

PFR: platelet-rich fibrin; SD: standard deviation; Min.: minimum; Max.: maximum. Source: Elaborated by the authors.

Regarding pressure, it was not observed a significant difference between treatments between the groups. In the malnourished group that received PRF, the mean was 186.4 mmHg and median 193.8 mmHg. In the group of fed animals, the average was 201.3 mmHg, and the median was 197.1 mmHg (Table 8).

**Table 8:** Global burst pressure.

		Pressure			*p*-value
Group	Treatment	Average	SD	Median	
Malnourished	Without PRF	157.5	112.1	173.1	0.867
Malnourished	With PRF	186.4	44.6	193.8	
Nourished	Without PRF	180.1	47.0	171.9	0.248
Nourished	With PRF	201.3	52.3	197.1	

PFR: platelet-rich fibrin; SD: standard deviation. Source: Elaborated by the authors.

When evaluating the average stained area representative of collagen, in the subgroup without PRF, the value was equal to 13,343 μm^2^ ± 3722.47 μm^2^. In the group that received PRF, the average was 14,839.25 μm^2^ ± 6,116.13 μm^2^, with no statistically significant difference (*p* = 0.2917) (Table 9).

**Table 9:** Collagen analysis in malnourished animals.

Without PRF (n = 8)	Collagen-stained area (μm^2^)	With PRF (n = 8)	Collagen-stained area (μm^2^)
HS-PRF 1	10,060.12	HC-PRF 1	18,870.88
HS-PRF 2	7,406.89	HC-PRF 2	12,549.88
HS-PRF 3	7,275.89	HC-PRF 3	10,760.92
HS-PRF 4	13,518.55	HC-PRF 4	10,757.14
HS-PRF 5	9,785.49	HC-PRF 5	13,267.87
HS-PRF 6	14,027.08	HC-PRF 6	18,695.46
HS-PRF 7	20,908.83	HC-PRF 7	20,890.40
HS-PRF 8	23,765.48	HC-PRF 8	16,777.49
Average#	13,343.00	Average#	14,839.25

# *p* = 0.2917;

PFR: platelet-rich fibrin. Source: Elaborated by the authors.

In malnourished animals without PRF, the area stained with reticulin reagent was smaller compared to animals that received PRF. The concentration of brown dye was predominant in this case, which characterizes a higher concentration of reticulin in this fragment. It can be observed that the mean area-stained representative of reticulin in the subgroup without PRF was equal to 13,165.94 μm2 ± 5,201.91 μm^2^, while in the group that received PRF it was 18,715.92 μm^2^ ± 5,229.54 μm^2^ (*p* = 0.040) (Table 10).

**Table 10:** Reticulin analysis in malnourished animals.

Without PRF (n = 8)	Collagen-stained area (μm^2^)	With PRF (n = 8)	Collagen-stained area (μm^2^)
HS-PRF 1	10,808.29	HC-PRF 1	11,471.69
HS-PRF 2	24,214.07	HC-PRF 2	24,504.26
HS-PRF 3	12,936.94	HC-PRF 3	21,643.47
HS-PRF 4	7,798.94	HC-PRF 4	18,722.40
HS-PRF 5	7,695.38	HC-PRF 5	17,565.56
HS-PRF 6	14,132.83	HC-PRF 6	15,600.31
HS-PRF 7	13,132.83	HC-PRF 7	26,511.66
HS-PRF 8	14,607.53	HC-PRF 8	13,708.03
Average#	13,165.94	Average#	18,715.92

# *p* = 0.040;

PFR: platelet-rich fibrin. Source: Elaborated by the authors.

## Discussion

The aim of this study was to evaluate the effect of PRF on the healing process of colic sutures in an animal model. We investigated the effects of PRF in rats with preserved nutritional status and in those with malnutrition. The choice to perform colotomy and colorraphy instead of a complete anastomosis was due to better obtaining a specimen for tests, as occurs with the cecum in rats. This segment was easily adapted to the burst pressure analysis device and showed no defect at its opposite end. The decision to use human PRF was due to the difficulty in obtaining a satisfactory quantity of blood from rats, especially malnourished ones. In malnourished rats, collecting autologous blood was not feasible, leading to the death of the two animals in the pilot project. Since they were very fragile animals, we decided to use human blood. Refined surgical techniques using high-quality sutures and tension-free anastomoses are the main factors for a successful anastomosis. New treatment modalities are needed to avoid anastomotic failure, especially in high-risk patients^7^.

García-Vásquez et al.^8^ demonstrated, in an experimental study in pigs submitted to intestinal ischemia, that the fibrin patch increases the duration of the healing process in the surgical bed, due to greater exposure to agents of the healing cascade. Alvarenga et al.^11^ analyzed the protective effect of mesenchymal cells derived from adipose tissue in an experimental model of high-risk colonic anastomosis in rats with induced chemical colitis underwent colectomy. In the group that received protection, there were an improvement in the immunomodulatory response and better healing results, compared to the control group that did not receive protection.

PRF is a new generation of platelet concentrate capable of stimulating the defense mechanism during wound healing^9^. Fibrin also plays a role as a natural guide for angiogenesis. It can be used to promote bone regeneration, graft stabilization, wound sealing, and hemostasis^10^.

During the feeding phase, there was a loss of four animals, two in each group of malnourished animals, totaling 10% of the total sample. In similar studies, mortality rates ranged between 0 and 20%. The similar losses were observed without detrimental effect on sample power^12^.

Studies on the use of PRF and other biomaterials in intestinal sutures are very limited. Most of them evaluate suture lines in terms of rupture pressure and the inflammatory process, emphasizing on elements of the extra-cellular matrix. This study found that, for malnourished rats that received PRF, the rupture did not occur at the suture line, but outside it, in all animals when compared to malnourished animals that did not receive the membrane. Therefore, it can be raised that, in situations of nutritional deficiency, reinforcing an intestinal suture with this biomaterial is beneficial.

In the field of dentistry/periodontics, the use of PRF has been widely studied. A study that evaluated a possible exacerbated inflammatory response caused by the membrane found that PRF can reduce the release of interleukin-1β and, at least partially, inhibit factors related to pyroptosis in macrophages induced by lipopolysaccharide in vitro. This reinforces the anti-inflammatory role of the compound, which possibly does not induce unfavorable antigenic action^13^.

For malnourished animals, colon healing was evaluated by four methods: integrity of the anastomosis, site of rupture, rupture pressure, and quantification of collagen and reticulin. In malnourished animals that received the PRF membrane, all ruptures occurred outside the suture line. In animals that did not receive PRF, the rupture occurred at the suture line in three animals, another three outside the suture line, and two animals presented dehiscence.

The influence of malnutrition on the healing of colic anastomoses has been discussed a lot. We demonstrated the negative effects of malnutrition on colonic anastomoses in rats, resulting in rupture for malnourished animals with PRF application, outside the suture line, as well as a greater percentage area of reticulin deposition in those animals that received PRF. Experimental studies confirm the beneficial effects of preoperative nutrition^14^. The present study focused on colon healing in malnourished rats, and we can argue that the significant difference in the weight of malnourished animals may be a risk factor for the failure of intestinal sutures.

Ikeuchi et al.^15^ demonstrated that most of ruptures occur in the anastomotic line if the analysis is carried out until the fourth postoperative day. Therefore, in this study, the seventh postoperative day was chosen for analysis of tensile strength to avoid ruptures outside the anastomotic site. Some authors describe that ruptures occurred possibly due to unnecessary dissection during specimen removal, weakening the colon wall beyond the anastomoses in rats that developed greater adhesion^16^. For this reason, in this study it was decided not to dissect the specimen close to the suture line to avoid this occurrence. The largest area of collagen was measured only in the malnourished groups, since there was no difference in the evaluations of the nourished groups for pressure and rupture location.

Gonçalves et al.^17^ demonstrated a large area occupied by type I (mature) collagen in animals receiving preoperative nutrition. Groups of malnourished animals contained a smaller area of type I collagen compared to controls. Due to these factors, the study indicates that animals in the group with greater nutritional attention showed better results in terms of the anastomoses healing process. Our results allow to infer that malnutrition locally compromises the healing of the anastomosis, which was documented by macroscopic evaluation and low anastomosis rupture pressures. Treatment with PRF can have a marked influence on these parameters and could avoid local complications of the anastomosis.

The animals in the group of malnourished rats treated with PRF (group IV) showed significantly fewer complications, when compared to the group of malnourished animals, in which PRF was not used (group III). Anastomotic rupture pressures were higher in animals treated with PRF in group IV. These data indicate that PRF prevents local complications and promotes and stimulates the anastomosis healing process, especially when this process is potentially compromised.

Animals in group IV showed better histological scores when compared to group III. Although the differences between the malnourished groups did not reach statistical significance for collagen assessment, it was achieved for reticulin measurement. These data indicate that the beneficial effect of PRF is more prominent when intestinal anastomosis healing is potentially compromised. The animals in groups I and II were not evaluated in the histological analysis because there was no statistically significant difference regarding the rupture site.

Histopathological evaluations of the animals’ anastomosis sites also showed complete dissolution of the PRF membrane from the intestinal surfaces. Furthermore, the use of PRF significantly reduced leaks in the suture line in malnourished animals, leading to the inference that the colonic wall became less resistant than the suture line itself, which was documented in the macroscopic evaluation. The application of PRF to the intestinal wall therefore prevents local complications and stimulates healing process in malnourished animals.

The observation of the point of greatest weakness of the intestinal wall outside the suture area in the malnourished group that received the membrane demonstrates that the suture line becomes more resistant than the intestinal wall itself. Although the rupture pressures were similar, it was possible to demonstrate that there was structural reinforcement in the sutured region, which was not evaluated in previous studies.

As limitations of this study, it can be said that the histological evaluation of the nourished groups could added valid comparative data. Likewise, the histological study could be more specific in terms of cellularity and types of collagens assessed. Moreover, malnutrition parameters could have been also assessed biochemically. Assessment at later postoperative stages could provide relevant data.

The preparation of PRF is very simple and does not require anticoagulant or bovine thrombin. This preparation could be done in the operating room during operations, and the PRF membrane could be applied immediately after its preparation in the intestinal anastomoses sites6. This study opens the field for others that can test PRF as an auxiliary substrate in the healing of fistulas, like perianal, recto-urethral or recto-vaginal fistulas. Its use in humans is safe and can be the target of research in the context of the gastrointestinal tract.

## Conclusion

The use of PRF membrane as reinforcement of intestinal sutures in malnourished rats can improve resistance in the synthesis area. On the other hand, there was no difference in intestinal burst pressure between the groups of nourished and malnourished rats, regardless of the application of PRF. The concentration of reticulin was significantly higher in malnourished animals that received PRF, while the concentration of collagen tended to be higher in rats that received the PRF membrane, which suggests enhancement of the healing process in animals with nutritional deficiencies.

A field for future studies remains open to test PRF as an auxiliary substrate in the healing of perianal, recto-urethral or recto-vaginal fistulas. Its use in humans is safe and can be the target of research in the context of the gastrointestinal tract.

## Data Availability

All data sets were generated or analyzed in the current study.
